# On the Presence of Albumen in the Urine

**Published:** 1849-05

**Authors:** 


### PATHOLOGY AND PRACTICE OF MEDICINE.

*On the presence of Albumen in the Urine.* By Dr. FINGER.—Since it has been determined by repeated observations that the presence of albumen in the urine occurs in other cases than in those of Bright's disease of the kidney, other signs have been advanced as peculiar to, and characteristic of, this disease—*e. g.* the diminution of urea in the urine, the occurrence of fibrinous casts of the urinary tabules in this fluid, the presence of urea and of uric acid in the blood and dropsical fluids. But none of these circumstances can be regarded as peculiar to Bright's disease alone. In the course of his inquiries into the subject of morbus Brightii, Dr. Finger examined the urine of about 600 patients, and frequently found albumen in it. Of 186 cases of tubercular disease, the urine contained albumen in 46, while in only 2 of this number was there evidence of Bright's disease. Of 88 cases of typhus, the urine was albuminous in 29, becoming so on the average from the sixteenth to the twenty-fifth day of the disease; in those of the 29 that recovered, the albumen disappeared from the urine as they became convalescent. 17 of the 29 died; the kidneys were in all perfectly healthy. Of 46 cases of puerperal fever, the urine contained albumen in 32; in 6 of these which died, and in which the urine had continued albuminous after the cessation of the lochial discharge, dissection shewed purulent peritonitis, the kidneys being healthy. Of 14 cases of carcinoma, the urine was albuminous in 6; examination after death discovered carcinomatous ulceration of the stomach in 3 of these, and of the uterus in the other 3; the albumen in the urine in the latter cases might have been due to this fluid being mixed with the discharge from the ulcerating surfaces. Of 33 cases of pueumonia, the urine contained albumen in 15; of these 6 died, and the kidneys in all were found healthy. Of 65 cases of intestinal catarrh, albumen was found in the urine of 8, 3 of whom died.

Dr. Finger believes that in the above cases, the albumen in the urine is not simply separated as such, but is probably a constituent of inflammation, lymph, or of pus, which has been formed in some part of the body, and brought by the circulation to the kidneys, whence it finds its way into the urine; for in many of the cases the formation of pus in some part was attended by the simultaneous appearance of albumen in the urine. In addition to the previously mentioned cases, Dr. F. found albumen in 2 out of 6 cases of chlorosis, in 2 out of 16 cases of acute rheumatism, in 1 out of 10 of intermittent, in 2 out of 14 of pleurisy, in 2 out of 6 of peritonitis, in 3 out of 16 of chronic bronchitis, and in 7 out of 18 of disease of the heart. A remarkable circumstance was observed in regard to the urine of two epileptics. One of the patients was a labourer, aged 32, who had been epileptic for years, but was otherwise in good health; in him, after each attack of epilepsy, a large quantity of albumen appeared in the urine, then gradually diminished, and in about 36 hours quite disappeared, until the next attack, when it again occurred in large quantity. The same phenomenon was observed in the other epileptic, a young girl 12 years of age, who was otherwise in good health.—*Ibid, from Osterreichische Medicinische Wochenschrift.*

